# First-Principles Study on the Structural and Electronic Properties of Monolayer MoS_2_ with S-Vacancy under Uniaxial Tensile Strain

**DOI:** 10.3390/nano8020074

**Published:** 2018-01-29

**Authors:** Weidong Wang, Chenguang Yang, Liwen Bai, Minglin Li, Weibing Li

**Affiliations:** 1 School of Mechano-Electronic Engineering, Xidian University, Xi’an 710071, China; cgyangxdu@foxmail.com (C.Y.); lwbaixdu@foxmail.com (L.B.); 2 Department of Mechanical Engineering, Northwestern University, Evanston, IL 60208, USA; 3 School of Mechanical Engineering and Automation, Fuzhou University, Fuzhou 350108, China; liminglin@fzu.edu.cn; 4 ZNDY of Ministerial Key Laboratory, Nanjing University of Science and Technology, Nanjing 210094, China; 5 McCormick School of Engineering and Applied Science, Northwestern University, Evanston, IL 60208, USA

**Keywords:** monolayer MoS_2_, S-vacancy, first-principles study, uniaxial tensile strain, structural property, electronic property

## Abstract

Monolayer molybdenum disulfide (MoS_2_) has obtained much attention recently and is expected to be widely used in flexible electronic devices. Due to inevitable bending in flexible electronic devices, the structural and electronic properties would be influenced by tensile strains. Based on the density functional theory (DFT), the structural and electronic properties of monolayer MoS_2_ with a sulfur (S)-vacancy is investigated by using first-principles calculations under uniaxial tensile strain loading. According to the calculations of vacancy formation energy, two types of S-vacancies, including one-sulfur and two-sulfur vacancies, are discussed in this paper. Structural analysis results indicate that the existence of S-vacancies will lead to a slightly inward relaxation of the structure, which is also verified by exploring the change of charge density of the Mo layer and the decrease of Young’s modulus, as well as the ultimate strength of monolayer MoS_2_. Through uniaxial tensile strain loading, the simulation results show that the band gap of monolayer MoS_2_ decreases with increased strain despite the sulfur vacancy type and the uniaxial tensile orientation. Based on the electronic analysis, the band gap change can be attributed to the π bond-like interaction between the interlayers, which is very sensitive to the tensile strain. In addition, the strain-induced density of states (DOS) of the Mo-*d* orbital and the S-*p* orbital are analyzed to explain the strain effect on the band gap.

## 1. Introduction

Since the discovery of graphene, 2D materials such as boron nitride (BN), molybdenum disulfide (MoS_2_), and tungsten disulfide (WS_2_) have become one of the hot topics in the scientific community because of the fantastic physical properties and promising applications of these materials in flexible electronic devices [1]. Among the 2D materials mentioned above, MoS_2_ is one candidate for flexible electronic devices because of its good mechanical and electrical properties. The bulk of MoS_2_ belongs to the space group *P*6_3_/*mmc*. The monolayer MoS_2_ can be viewed as a cleaved form of the (001) surface of bulk MoS_2_ [2]. It is different from the structure of graphene in that it has three atomic layers composed of one Mo layer plus two S layers on both sides, which are held together by van der Waals interactions [3]. During its application, flexible electronic material will inevitably suffer bending deformations. Up to now, the preparation of ultra-thin MoS_2_ films, even a monolayer MoS_2_ structure on a substrate, can be successfully realized by many methods such as electron irradiation and chemical vapor deposition (CVD) [4]. However, vacancy defects can be produced with high density on the MoS_2_ surface, and this can influence the physical properties and band structures. Therefore, it is very important to explore the influence of these factors on its mechanical and electrical properties.

Recently, much interest has been focused on the strain or vacancy effect on the properties of MoS_2_ from both experimental [5,6,7,8] and theoretical [9,10,11,12] aspects. Cooper et al. [5] explored the nonlinear elastic properties by indenting suspended circular MoS_2_ membranes with an atomic force microscope. Bertolazzi et al. [6] found that the in-plane stiffness of monolayer MoS_2_ is 180 ± 60 N·m^−1^, which corresponds to an effective Young’s modulus of 270 ± 100 GPa. Wang et al. [7] observed a strong shrinkage of the band gap with a rate of 48 meV/% of strain for monolayer MoS_2_. Santosh et al. [8] examined by scanning tunneling microscopy the possible defect structures and their impact on the electronic properties of the MoS_2_ monolayer. In addition to the experimental work, there has been a lot of work on MoS_2_ such as molecular dynamics (MD) simulations [9,10] and first-principles studies [2,5,11,12]. Wang and his co-authors [9] used nanoindentation simulations to find Young’s modulus of 280 ± 21 GPa. Li et al. [10] investigated the effect of V_MoS3_ point defects on the elastic properties of monolayer MoS_2_ sheets by using the classical MD simulation with reactive empirical bond-order (REBO) potential. Cooper et al. [5] applied density functional calculations based on local density approximation (LDA) and estimated Young’s modulus of MoS_2_ nanosheets equal to 210 GPa. Lu et al. [2] explored through first-principles calculations the tensile or compressive axial strain in different charities and found different effects on the band gap. Recently, much work has been done to calculate the properties of strained MoS_2_ using the DFT method. For instance, Spirko et al. [11] investigated the defect structure of monolayer MoS_2_, Defo et al. [12] proved the effect of strain on band gap regulation by applying compressive and tensile strain to single layer MoS_2_, and Maniadaki et al. [13] calculated the band gap of transition metal dichalcogenide monolayers and found it to decrease while dielectric constants increased for heavier chalcogens X. In addition, Coehoorn et al. [14] investigated the defect structure of monolayer MoS_2_ using DFT calculations and Lebègue et al. [15] calculated the electronic structure of MoS_2_ using the ab-initio method. All of these studies have many important conclusions; however, there is little research about the influence of vacancy on the structural and electronic properties of MoS_2_ when its energy gap is controlled by uniaxial tensile strain. Therefore, it is necessary to investigate the influence of these two factors comprehensively.

In the present work, we investigate the structural and electronic properties of monolayer MoS_2_ with S-vacancy defects under tensile strain loading by using first-principles calculations. The effect of tensile strain on the density of electronic states is revealed. The mechanical properties of monolayer MoS_2_ are weakened due to the existence of defects, and the band gap of monolayer MoS_2_ undergoes a descending trend with increasing strain, which means a semiconductor with a lower band or metal transition can be achieved with the application of strain.

## 2. Method

First-principle calculations were carried out using density functional theory (DFT) calculations in the projector augmented-wave (PAW) pseudopotential as implemented within the Vienna Ab-initio Simulation Package (VASP) [16,17]. The electronic exchange-correlation potential was used by the Perdew–Burke–Ernzerhof (PBE) flavor [18]. Electronic kinetic-energy cutoff was set to 450 eV [19]. The Brillouin zone integration was carried out by using a 12 × 12 × 1 k-mesh according to the Monkhorst–Pack scheme. For the physical modeling of monolayer MoS_2_, the supercell kept periodical boundary conditions in the *x-* and *y*-directions, as well as during DFT simulations, and a large enough vacuum region in the *z*-direction (20 Å) was set to prevent inter-layer interactions. 

**Figure 1:**
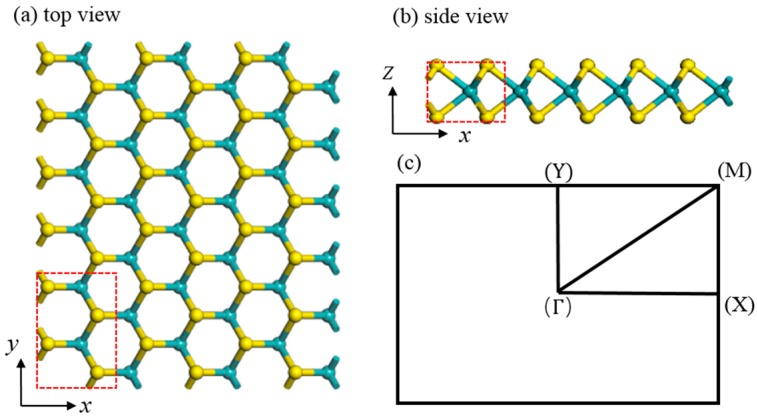
The 3 × 3 × 1 supercell structure of a perfect molybdenum disulfide (MoS_2_) monolayer: (**a**) top view and (**b**) side view. The original cell is delineated with the red dashed line frame and the first Brillouin zone is described (**c**).

The structure was fully relaxed with an energy convergence of 1.0 × 10^−6^ eV. The atomic Hellman–Feynman force was minimized to less than 0.01 eV/Å. Especially, a rectangle supercell was chosen in our calculations, and the red dashed frame indicates the original cell that involves four Mo atoms and eight S atoms. The 3 × 3 × 1 supercell structure of a perfect monolayer MoS_2_ from top view in *x*-and *y*-directions is shown in Figure 1a and its side view is given in Figure 1b. The original cell is delineated with a red dashed line frame including 2 × 1 primitive cells. The first Brillouin zone is described in Figure 1c for this model.

**Figure 2:**
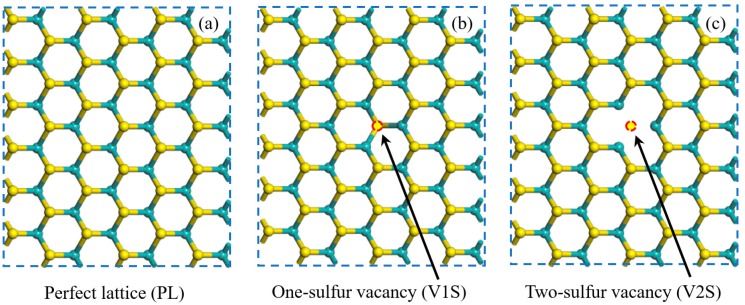
The top view of the atomic configurations of monolayer MoS_2_: (**a**) perfect lattice (PL) structure, (**b**) one-sulfur vacancy (V1S) structure, and (**c**) two-sulfur vacancy (V2S) structure.

In the present study, the monolayer MoS_2_ with a sulfur-vacancy defect under different tensile strains along the *x-* and *y*-directions was investigated, respectively. As shown in Figure 2a,c, we considered three MoS_2_ lattice structures: a perfect lattice (PL), a one-sulfur vacancy (V1S) lattice, and a two-sulfur vacancy (V2S) lattice. To further understand the vacancy defect, we calculated the vacancy formation energy for the lattice with a vacancy in Figure 2b,c. The vacancy formation energy *E*_f_ is defined as *E*_f_ = *E*_d_ − *E*_p_ + *nE*_i_ where *E*_d_ is the total energy of the vacancy lattice, *E*_p_ is the corresponding perfect lattice, *n* is the number of atoms, and *E*_i_ is the chemical potential energy of the S atoms. We calculated the vacancy formation energies of V1S and V2S to be 6.59 eV and 13.08 eV, respectively. In addition, we calculated the Mo vacancy formation energy, which was much higher than the S vacancy, indicating that the S vacancy is more easily produced. The uniaxial tensile strain was simulated by enlarging the *x* and *y* cell lattice parameters, respectively, to a fixed larger value; then, the atomic positions were fully optimized. It is worth noting that in the original version of VASP, it cannot be achieved that a lattice parameter is fixed while other lattice constants are optimized. For this reason, we complied with the original VASP source code to enable the realization of this function.

## 3. Results and Discussion

### 3.1. Structural Properties

**Figure 3:**
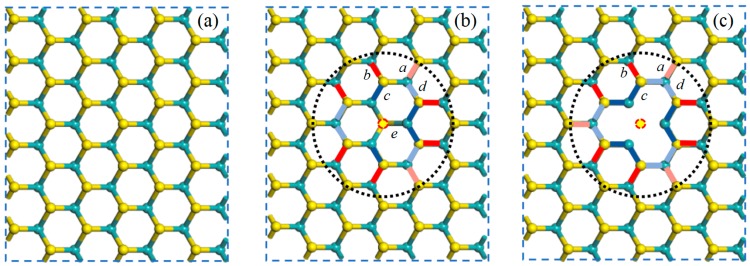
The atomic structures of PL (**a**), V1S (**b**), and V2S (**c**) in a monolayer MoS_2_. The bonds in the black dotted circle are colored according to the decrease (blue) or increase (red) in the bond length. The effects of a darker color are stronger than those of the lighter color.

In order to find the equilibrium lattice constant, full relaxations were conducted on the three models in Figure 2a–c. The lattice constant is 3.166 Å, the bond length of Mo–S and S–S are 2.415 Å and 3.131 Å, respectively, which agrees well with the available numerical results [20]. Figure 3 shows the atomic structures of the one-sulfur vacancy (V1S) and the two-sulfur vacancy (V2S) in monolayer MoS_2_. The bonds in the black dotted circle are colored according to a decrease (blue) or increase (red) in the bond length. The effects of a dark color are stronger than those of the light color. 

Compared to the perfect supercell model, there is little change in the lattice constant in the supercell containing vacancies after relaxation, indicating that a small amount of vacancy does not produce a significant impact on the crystal structure; however, the bond length around the vacancy is slightly different. Table 1 lists the lengths of the bonds of V1S and V2S of monolayer MoS_2_ before and after structure relaxation compared with perfect MoS_2_ in which *a*, *b*, *c*, *d*, and *e* represent the distance between the two adjacent Mo–S atoms in the sandwich layer around the vacancy. It is intuitively clear that ions surrounding S vacancies appear with slight inward relaxation because of the absence of sulfur atoms. For S vacancies in Figure 3b,c, the inward relaxations of the nearest Mo ions are larger than those of the other Mo ions [21]. The phenomenon can be explained by the redistribution of electrons. Compared with perfect MoS_2_, the adjacent S–Mo bond change is slightly larger than the sub-adjacent one, which conforms to the lattice distortion of a point defect. 

**Figure 4:**
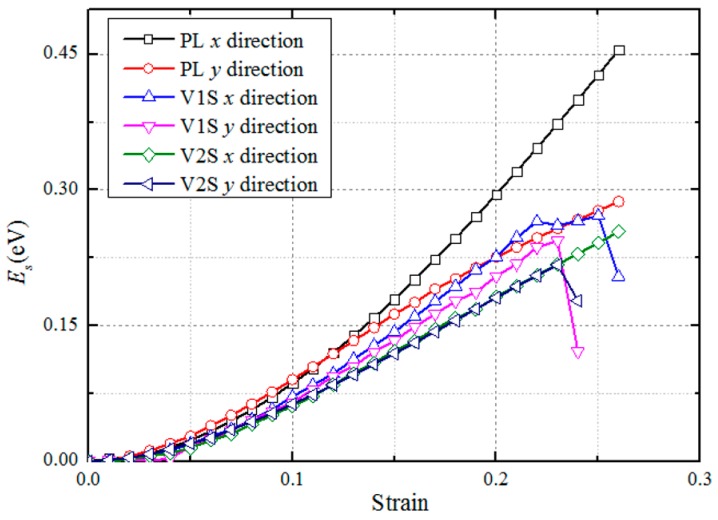
The per-atom strain energy *E_s_* of PL, V1S, and V2S monolayer MoS_2_ sheet versus tensile strain in the *x-* and *y*-directions.

To calculate Young’s modulus (*E*) and ultimate strength (*σ*_max_), we applied the uniaxial tensile strains on three models in the *x*- and *y*-directions, respectively. The applied strains in the *x*- and *y*-directions can be defined by Equation (1) [22]:

(1) $$\epsilon_{x}=\frac{a-a_{0}}{a_{0}}\;\mathrm{and}\;\epsilon_{y}=\frac{b-b_{0}}{b_{0}},$$

where *a* and *b* are the lengths of the supercell in the *x*- and *y*-directions after equilibrium and the lattice constants are *a*_0_ = 16.5672 Å and *b*_0_ = 19.1307 Å. The strains (*ϵ*) range from 0.00 to 0.30 with an increment of 0.01 in each step for three deformation cases. There are 90 ab initio DFT calculations in total. The per atom strain energy (*E_s_*) with tensile strain for PL, V1S, and V2S sheet in the *x*- and *y*-directions are shown in Figure 4. For small strains, *E*_s_ goes up with increased uniaxial tensile strain, while the influence of both uniaxial tensile orientation and sulfur vacancy types on *E*_s_ are very small. Once the strain goes beyond ~23%, *E*_s_ goes up with an increase in strain in the *x*-direction but slows in the *y*-direction. These strain–energy curves suggest the anisotropic properties of monolayer MoS_2_ with the tensile strain direction [23].

**Figure 5:**
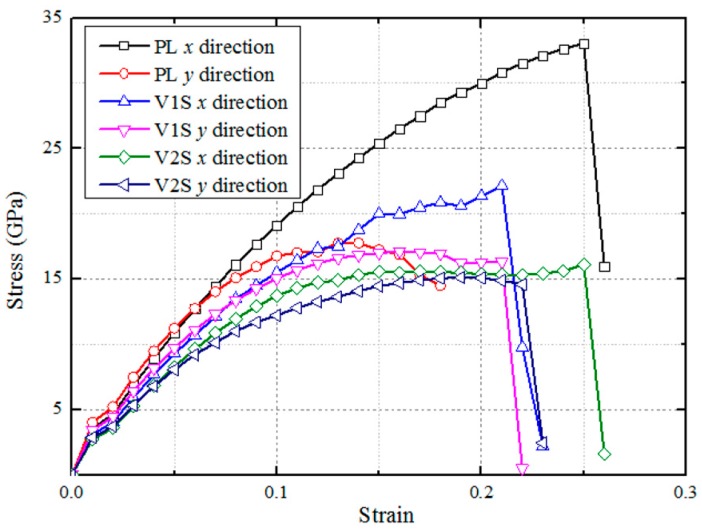
The variations of stress for the PL, V1S, and V2S monolayer MoS_2_ sheets versus the uniaxial tensile strain in the *x*- and *y*-directions.

Young’s modulus can be derived through the second derivative of the strain energy with respect to the strain and can be expressed by Equation (2) [24]:

(2) $$E=\frac{1}{V_{0}}\frac{\partial^{2}U}{\partial \epsilon^{2}}{|}_{\epsilon =0}$$

where *V*_0_ is the volume of monolayer MoS_2_, the thickness of it is defined as 6.145 Å, and *U* is the strain energy calculated by subtracting the total energy of the strained system from the equilibrium state. As shown in Figure 5, the strain–stress curves are plotted for the PL, V1S, and V2S monolayer MoS_2_ sheets, respectively. The tensile orientation effect on Young’s modulus is so slight that it can be neglected at small strains; however, the effect becomes apparent when the strain increases.

From Figure 5 it can be found that along the *x*-direction both the maximum stress and its corresponding strain are higher than those along the *y*-direction. The ultimate strength for a perfect lattice in the *x*-direction is larger than that in the *y*-direction [6]. In addition, the S-vacancy defects reduce the energy of the system. Compared with the PL MoS_2_ sheet, the S-vacancy MoS_2_ sheets reach the yielding point more quickly and, moreover, the yielding point of *x* is higher than the *y*-direction. In addition, for the V1S case, the symmetry is destroyed due to the sulfur vacancy and the oscillation occurs at the yielding point in the *x*-direction until the failure point is reached. In contrast, V2S reaches steady growth after reaching the yielding point until it reaches a point of failure. As shown in Table 2, the existence of a vacancy lowers the strength and stiffness of the material. The effects of orientation and sulfur defects on Young’s modulus and ultimate stress are not obvious. Moreover, Bertolazzi et al. have approximately obtained Young’s modulus of monolayer MoS_2_, which is equal to 270 ± 100 GPa [4]; Castellanos–Gomez et al. calculated the average of monolayer molybdenum disulfide to be around 330 ± 70 GPa using atomic force microscopy (AFM) [25]. Therefore, the simulation results of Young’s modulus in the present study agree well with the experimental data.

### 3.2. Electronic Properties

**Figure 6:**
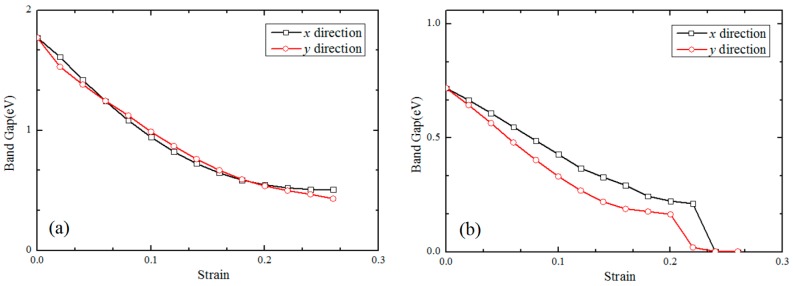

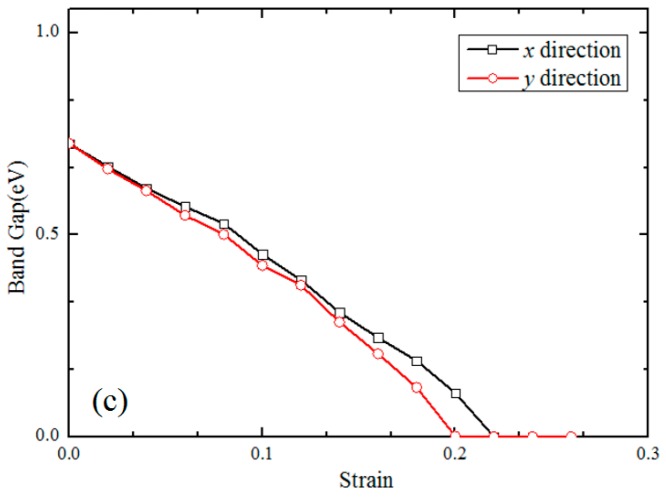
The band gaps of the PL (**a**), V1S (**b**), and V2S (**c**) monolayer MoS_2_ variation with the tensile strain in the *x*- and *y*-directions.

Strain modulation has been commonly used in low-dimensional systems to tune the electronic structures and band gaps. We further calculated the band gap of monolayer MoS_2_ sheets with two different types of sulfur-vacancy defects influenced by strain in the *x*- and *y*-directions, as shown in Figure 6. It can be found that the band gaps of the three cases—including PL, V1S, and V2S—decrease gradually as the tensile strain increases. The tendencies in the *x*- and *y*-directions are basically the same; the only difference is that the band gap of the monolayer MoS_2_ with sulfur vacancy defects will be reduced to zero. As shown in Figure 6, the calculated direct band gap for perfect monolayer MoS_2_ is about 1.77 eV at zero strain, which is consistent with the experimental value (1.90 eV [26]) and theoretical values (1.80 eV [27], 1.70 eV [28]). Both the V1S and V2S monolayer MoS_2_ sheets behave as semiconductors with band gaps of 0.72 eV and 0.73 eV, respectively. The reason why they changed a lot compared to the perfect one (PL) is mainly that the sulfur vacancy defect makes the original band gap impure.

**Figure 7:**
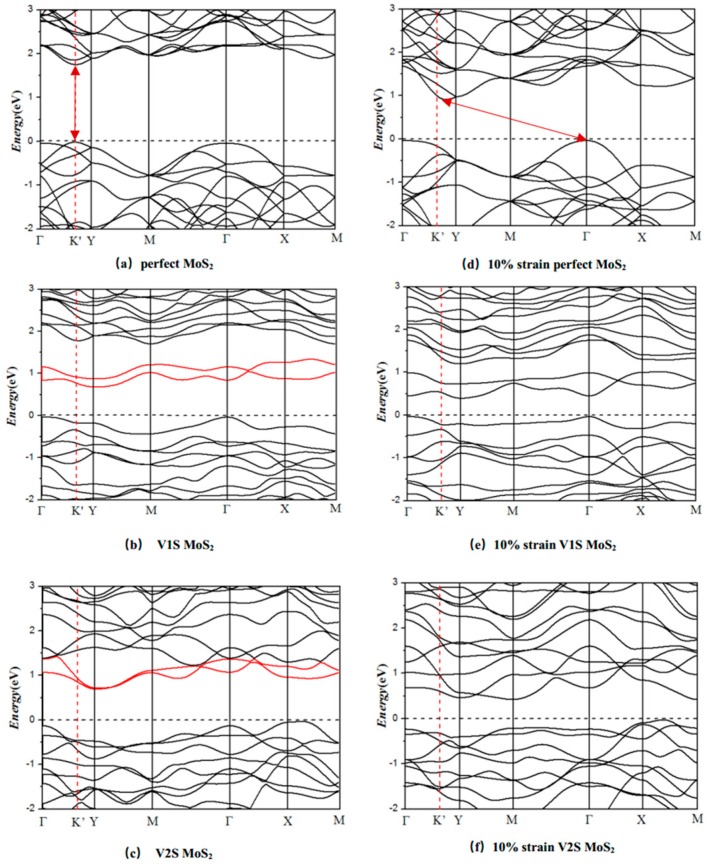
The band structure of the PL (**a**), V1S (**b**), and V2S (**c**) monolayer MoS_2_ sheet at natural state (zero strain) and under 10% uniaxial tensile strain in the *x*-direction (**d**–**f**). The dotted line represents the Fermi energy level.

Considering the tensile strain, the electronic property of the perfect and S-vacancy MoS_2_ are revealed through investigating their band structures, which are shown in Figure 7. From the band structure, it is found that the presence of sulfur vacancies leads to the introduction of new energy bands near the Fermi level and the hydrogen adsorption on the S-vacancies may be the reason for it. Because of the introduction of the sulfur vacancy, the direct band gap of monolayer MoS_2_ has been changed into an indirect band gap, which is due to the contraction of the neighboring atoms of S-vacancy; a local state or transition state occurs between the VBM and CBM [10,19]. A defect energy band is introduced (red line). The direct band gap of monolayer MoS_2_ has been changed into the indirect band gap, which is due to the contraction of the neighboring atoms of the S-vacancy. A local state or transition state occurs between the VBM and CBM [10,19].

With an increase in the tensile strain, the conduction band minimum (CBM) moves down but the valence band maximum (VBM) does not visibly change; the CBM point moves from the Γ point, which leads to the band gap changing from direct into indirect. At the same time, the influence of orientation is not obvious, so the *x*-direction is taken as an example.

**Figure 8:**
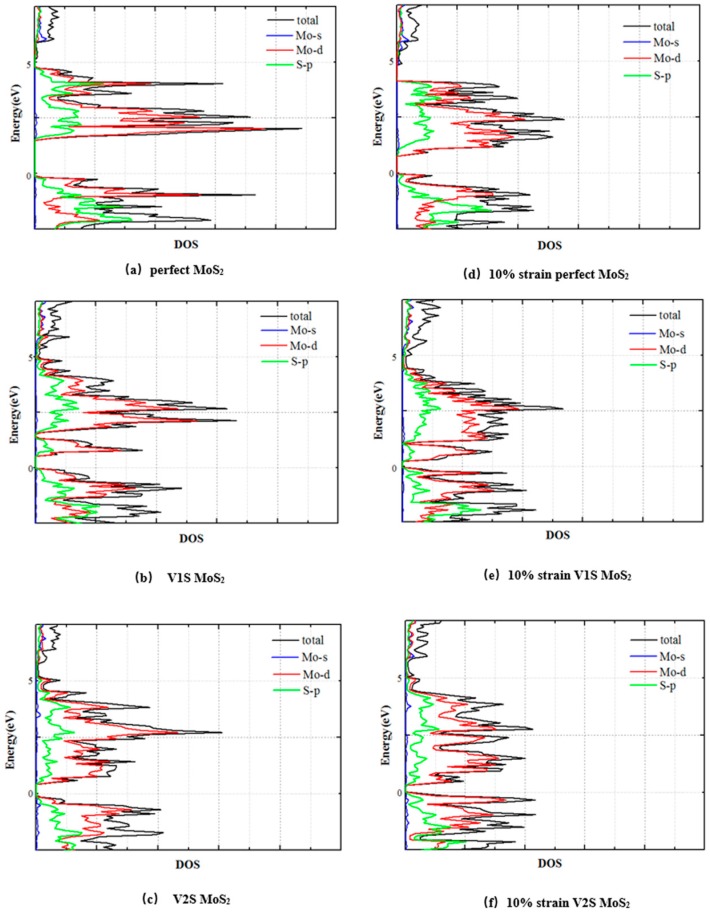
The electron density of states (DOS) for the PL (**a**), V1S (**b**) and V2S (**c**) monolayer MoS_2_ sheet at natural state (zero strain) and under 10% uniaxial tensile strain in the *x*-direction (**d**–**f**). Zero energy represents the Fermi energy level.

Figure 8 shows the density of states (DOS) of the PL, V1S, and V2S monolayer MoS_2_ and their 10% strain under the uniaxial tension in the *x*-direction. For the perfect monolayer MoS_2_, as well as one with defects, CBM and VBM are mainly composed of S-*p* orbitals and Mo-*d* orbitals. At the same time for V1S and V2S defects of MoS_2_, as shown in Figure 8a–c, taking V1S as an example, the existence of a transition state that reduces the band gap is mainly due to the interaction above the Fermi level of the S-*p* and Mo-*d* orbitals. The main reason for the transition state is that the sulfur vacancy causes contraction of the Mo–Mo bond around the defect. At the same time, the highly symmetric bond structure of S–Mo–S forms the role of a weak π bond-like interaction, which is extremely sensitive to strain, resulting in a significant change in the energy band structure once the strain is applied. As shown in Figure 8d–f, after the strain is applied, the DOS around the Fermi level changes significantly and the variation of the S-*p* orbit is more obvious than that of the Mo atomic orbital. The PL MoS_2_ sheet presents a direct band gap structure and there is no other energy level in the band gap. Compared with the PL electronic structure, the presence of S-vacancies makes the three Mo atoms around the vacancies have six Mo-*d* orbital electrons that are not bonded with the S atoms, which are acting as electron donors in the defect system. The excess Mo atoms’ Mo-*d* orbit electrons fill into these defect levels. The analysis holds that the two levels are more likely to belong to the acceptor’s energy level. 

**Figure 9:**
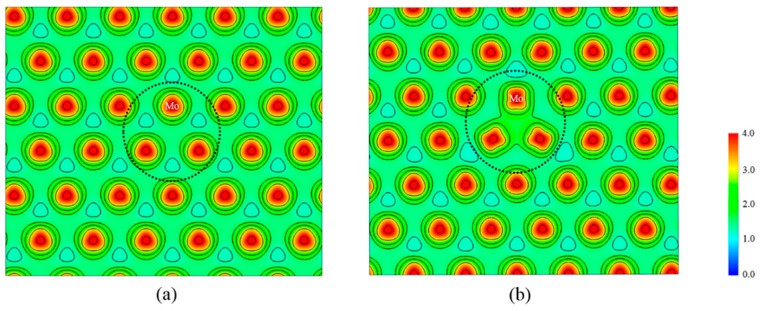
The electron density of a Mo layer. (**a**) The Mo bonding in intrinsic monolayer MoS_2_. (**b**) The Mo bonding in two-sulfur vacancy of monolayer MoS_2_.

We also examined the electron densities of the Mo layer, which can reflect changes in the chemical bonds around the vacancies. Figure 9 compares the electron density of the Mo layer between intrinsic and sulfur vacancies of the monolayer MoS_2_. It can be seen from the figure that in the absence of sulfur in a symmetrical position in the MoS_2_ structure, the electrons near the vacancy are concentrated mainly in three adjacent Mo atoms. The high density of electrons gathered makes the three Mo atoms almost together. Also, a strong coulomb attraction is produced to the nearest Mo atom or S atom and that leads to the S–Mo bonds in the foreground becoming smaller. Compared with the perfect state, the electron distribution near the vacancy area is obviously localized and should be related to the formation of the defect level. 

## 4. Conclusions

In summary, through DFT calculations, the structural and electronic properties of monolayer MoS_2_ with one or two sulfur vacancies have been investigated systematically under uniaxial tensile strain in the *x*- and *y*-directions. Regarding structural properties, it was found that the existence of vacancies can weaken the stiffness and strength of the MoS_2_ sheet. Young’s modulus and strength of perfect MoS_2_ have been determined to be 315 GPa and 33.2 GPa, respectively, in the *x*-direction; the one-sulfur vacancy MoS_2_ reduces to 272 GPa and 21.9 GPa, respectively. Differences exist in the weakening effect in different uniaxial tensile loading directions (i.e., the *x*- and *y*-directions). 

Besides, an analysis of the electronic properties shows that both of the two vacancy types behave as semiconductors and that their band gap is much smaller than perfect monolayer MoS_2_ because of the impurity. The band gap of perfect monolayer MoS_2_ is about 1.77 eV while the one and two sulfur-vacancy MoS_2_ are 0.72 eV and 0.73 eV. The strains also have a huge impact on the band gap of defected MoS_2_ sheets because of the relaxation near the vacancy. By comparing the electronic structures with that of perfect MoS_2_, the existence of vacancy defects has obvious effects on the electronic structures of monolayer MoS_2_, especially for the high-energy region of the conduction band density of state. These effects may be related to the defect energy levels introduced by the vacancy defects. Above all, our study explored MoS_2_ subjected to external uniaxial tensile strain, which can present band gap changes to tune its resistance, which makes it applicable in MoS_2_ piezo-resistive sensors. At the same time, the effects of sulfur vacancies on the mechanical properties and the band gaps of the material have been studied to determine the measuring range of the sensor and the interference from the vacancy.

## Figures and Tables

**Table 1:** The length of bonds of V1S and V2S of the monolayer MoS_2_ before and after structure relaxation compared with the perfect MoS_2_ (Unit: Å).

Model Type	*a*	*b*	*c*	*d*	*e*
PL	2.415	2.415	2.415	2.415	2.415
V1S	2.430	2.450	2.398	2.402	2.371
V2S	2.428	2.453	2.400	2.405	/

**Table 2:** Young’s modulus (*E*) and maximum strength (*σ*_max_) of monolayer MoS_2_ for a perfect film and one-sulfur (V1S) and two-sulfur (V2S) vacancies.

	*E* (GPa)		$\sigma_{\max}$ (GPa)	
	*x*	*y*	*x*	*y*
**Perfect**	315	335	33.0	17.7
**V1S**	272	290	22.1	17.1
**V2S**	227	241	16.1	15.1
